# Study protocol of a randomized clinical trial evaluating the effectiveness of a primary care intervention using the Nintendo™ Wii console to improve balance and decrease falls in the elderly

**DOI:** 10.1186/s12877-015-0178-x

**Published:** 2016-01-12

**Authors:** Pilar Montero-Alía, Laura Muñoz-Ortiz, Mercè Jiménez-González, Carla Benedicto-Pañell, Salvador Altimir-Losada, Yolanda López-Colomer, Josep Prat-Rovira, Joan Francesc Amargant-Rubio, Sheila Mendes Jastes, Ana Moreno-Buitrago, M. Carmen Rodríguez-Pérez, Cristina Teixidó-Vargas, José Luís Albarrán-Sánchez, Anna Candel-Gil, Domènec Serra-Serra, Juan José Martí-Cervantes, Carlos Andrés Sánchez-Pérez, Lidia Sañudo-Blanco, Sònia Dolader-Olivé, Pere Torán-Monserrat

**Affiliations:** Primary Healthcare Research Support Unit Metropolitana Nord, Institute of Primary Care Research (IDIAP) Jordi Gol, Calle Major 49-53, 08921 Santa Coloma de Gramenet, Spain; Primary Healthcare Centre Riera (Mataró 1), Catalan Health Institute, Calle Ronda Prim 35, 08302 Mataró, Spain; Primary Healthcare Centre Rocafonda (Mataró 3), Catalan Health Institute, Ronda Pintor Rafael Estrany 24, 08320 Mataró, Spain; Hospital Universitari Germans Trias i Pujol, Carretera de Canyet s/n, 08916 Badalona, Spain; Residència de Gent Gran del Institut Català d’Assistència i Serveis Socials (ICASS), Gatassa 43, 08303 Mataró, Spain; Primary Healthcare Centre Gatassa (Radiologia), Catalan Health Institute, Camí del Mig 36, 08303 Mataró, Spain; Primary Healthcare Centre El Masnou, Catalan Health Institute, Calle de Sant Miquel 125, 08320 El Masnou, Spain; Faculty of Economics and Business, University of Barcelona, Avenida Diagonal 690, 08034 Barcelona, Spain; Primary Healthcare Centre Ronda Cerdanya (Mataró 5), Catalan Health Institute, Calle del Vallès 37, 08304 Mataró, Spain

**Keywords:** Postural balance, Aged, Randomized controlled trial, Intervention studies, Accidental falls, Primary Health Care, Virtual reality training

## Abstract

**Background:**

Balance alteration is a risk factor for falls in elderly individuals that has physical, psychological and economic consequences. The objectives of this study are to evaluate the usefulness of an intervention utilizing the Nintendo™ Wii console in order to improve balance, thereby decreasing both the fear of falling as well as the number of falls, and to evaluate the correlation between balance as determined by the console and the value obtained in the Tinetti tests and the one foot stationary test.

**Methods/Design:**

This is a controlled, randomized clinical trial of individual assignment, carried out on patients over 70 years in age, from five primary care centers in the city of Mataró (Barcelona). 380 patients were necessary for the intervention group that carried out the balance board exercises in 2 sessions per week for a 3 month period, and 380 patients in the control group who carried out their usual habits. Balance was evaluated using the Tinetti test, the one foot stationary test and with the console, at the start of the study, at the end of the intervention (3 months) and one year later. Quarterly telephone follow-up was also conducted to keep track of falls and their consequences.

**Discussion:**

The study aimed to connect the community with a technology that may be an easy and fun way to assist the elderly in improving their balance without the need to leave home or join rehabilitation groups, offering greater comfort for this population and decreasing healthcare costs since there is no need for specialized personnel.

**Trial registration:**

Current Control Trial NCT02570178.

## Background

Falls and their consequences are a major health problem in the elderly population, due to both the potential physical injuries (contusions, erosions, breaks) as well as the subsequent psychological problems (fear of falling, loss of self-confidence, loss of autonomy and decreased quality of life) [1], and also have major economic repercussions (hospitalizations, institutionalizations and even death) [2]. Over one year, 30 % of the adults over the age of 65 suffered from at least one fall [3]. Suffering from a fall is a risk in itself for subsequent falls.

Altered balance is one of the greatest risk factors for falls in the elderly. A worsening of balance occurs with aging as a result of physical limitations and a deteriorated peripheral sensory system. Obesity and a sedentary lifestyle have also been related to poor balance [4]. Considering the current increase in life expectancy, it is necessary to prevent the loss of balance in this aging population.

The most effective classical interventions in the prevention of falls consist of physical exercise, through improved balance, strength and walking practice [5], with the most effective treatments consisting of balance improvement based only on muscular strength [6]. Doing exercise in supervised groups, practicing Tai-Chi and participating in exercise programs on an individualized basis in the home have also been proven effective [3, 7]. Individual interventions have been found to be more effective than group interventions, suggesting an increased economic cost.

In a clinical trial, Robertson [8] demonstrated the economic benefits achieved with a home physiotherapy program for elderly individuals over the age of 80, with the number of falls being decreased as well as the subsequent hospital expenses derived from the same. These programs are limited by costs that may be incurred by the need for specialized personnel and their access which is restricted to a small population group.

Studies on the efficiency of exercises carried out using new technologies are based on bio-feedback. Bio-feedback is considered to be the best technique for working and learning new motor skills and for influencing the self-perception of effectiveness [9, 10].

The Nintendo™ Wii console offers the possibility of applying an exercise program to work on balance [11] and thereby improve walking. It is a low cost technology that may be applied in an individual or group basis, in a recreational manner, improving the perception of self-sufficiency. It also offers the advantage of not requiring a community rehabilitation program that is limited in time, not requiring professionals to lead the exercises, and not requiring specific facilities, thereby offering its users economic savings and increased convenience. According to the EXCELL [12] study and our clinical experience dedicated to determining the viability of the project, we believe that this tool may be well-accepted by the elderly.

The Wii Balance Board™ is a scale that allows for the calculation of the center of gravity (according to the distribution of weight on the board) as well as the percentage of balance based on a test called the lame duck, where the individual should stand as long as possible on one leg in the middle of the scale, supporting themselves on an object or person if necessary in order to facilitate the measurement [13].

Diverse studies suggest that there is a strong correlation between the Wii Balance Board™ and the so called gold standard laboratory platforms [14–16]. These platforms are appropriate for carrying out laboratory experiments but not for studies carried out in the clinical environment, due to their elevated cost and the difficulty of their transport and handling.

Increasing evidence suggests that training carried out with virtual reality and video games may improve balance in elderly individuals [12, 17] although the majority of the studies use small sample sizes, lack posterior follow up or have a very short follow up period and do not offer results on relapses [18].

Few clinical trials have been published on the efficiency of standardized, marketed videogames applied in clinical and rehabilitation environments. Morone et al. [19] carried out a clinical trial with patients in a sub-acute stroke stage in which the best balance results were obtained when associating conventional physiotherapy with training using the Nintendo™ Wii console.

The prevention of falls deserves a sufficient number of accessible programs that use as few healthcare resources as possible.

The main objectives of our study are to improve balance and decrease the number of falls in elderly individuals through a balance training carried out using a console. This project allows us to connect the community to an advanced technology that may be an easy form of assistance, without the need to leave home or join a rehabilitation group. Aiming to improve balance and reduce the number of falls in a fun and economic way is an ambitious goal of primary care for this ever growing population.

## Methods/Design

### Hypothesis

The balance exercises of the Nintendo™ Wii console may improve the balance of elderly individuals over the age of 70, reducing their fear of falls and reducing the number of falls suffered by this population.

We believe that there may be a strong correlation between the balance determined by the console and the Tinetti tests and the one footed stationary test in individuals over the age of 70.

The console shall be well accepted by the elderly individuals and they shall be satisfied with the exercise program used.

### Objectives

The principle objective is to evaluate the effectiveness of an intervention carried out using the Nintendo™ Wii console to improve balance in individuals over the age of 70.

The specific objectives are as follows:

- To evaluate the number of falls during the year of the intervention in the two study groups (intervention group and control group) and their consequences.
- To evaluate the correlation of balance determined by the console and the values obtained in the Tinetti tests and the one footed stationary test in individuals over the age of 70.
- To evaluate fear of falling before and after the intervention.
- To evaluate the acceptability of the console and the level of satisfaction of the elderly individuals in regards to the exercise program used.

### Design

A randomized, controlled clinical trial in parallel groups comparing individuals receiving balance training with the Nintendo™ Wii console with a control group that only received the standard practices.

The study is carried out according to the CONSORT guidelines. The study protocol is available on clinicaltrials.gov under the code NCT02570178 since July 2nd, 2015.

### Study subjects

Individuals aged 70 years or older, who are able to walk, attended to in any of the five centers of primary care of the city of Mataró (Barcelona).

### Inclusion criteria

Individuals aged 70 or older, of both genders, with the ability to walk, with or without technical assistance, who are available for a one year period and who agree to participate in the study, signing an informed consent form.

### Exclusion criteria

Home-bound patients, individuals who are already receiving rehabilitation treatment on walking, those with moderate cognitive deterioration (Pfeiffer ≥5), terminally ill patients, individuals who do not have a telephone, those with communication difficulties: cognitive and/or sensory deterioration, language barriers.

### Sample size

The variable used to calculate the sample size is based on the score of the sub-scale of the Tinetti’s stationary balance test which ranges from 0 to 16 points. Accepting an alpha risk of 0.05 points and a beta risk of below 0.20 in a bilateral contrast, 380 subjects are needed for the control group and 380 are needed for the intervention group in order to detect a difference that is equal or superior to 0.5 points in the sub-scale of Tinetti’s stationary balance test. The common standard deviation is assumed to be 2.2. This sample size will also allow for the detection of a difference that is equal or greater than 1 point in the Tinetti test (stationary balance sub-scale + walking balance sub-scale), and also permits detection of a correlation between the balance determined by the console and that of Tinetti’s test at a minimum of 0.12. A loss ratio has been estimated in the follow up of 20 %.

### Study variables

The following variables are included in both groups (control and intervention):

- Main variables:
  - Percentage of balance calculated by the Nintendo™ Wii console: Conducting the lame duck test which calculated how the center of gravity moves when the individual is on one foot for a maximum of thirty seconds. High percentages indicate good balance [13].
  - Balance calculated by the Tinetti Test: Evaluation of stationary balance (from 0 to 16 points) and evaluation of walking balance (from 0 to 12 points), with a maximum total of 28 points being the best balance [20].
  - Balance calculated by the one footed stationary test: Calculates the time a person can stand on one foot without assistance. The longer the time, the better the balance [20].
  - Fear of falling. The Short FES-I (Falls Efficacy Scale) looks at the probability of falling in seven everyday situations. Scoring ranges from 7 to 28 points (high scores indicate a greater fear of falling) [21].
- Secondary variables:
  - Number of falls. Record of the history of falls from the year prior to the study and prospective record of falls over the entire year of study as well as their consequences. A fall is considered to be any involuntary event leading to a loss of balance and the striking of the body to the ground or any other firm surface that stops it.
  - Functional capacity. Lawton and Brody scale that evaluates the capacity to carry out everyday situations (score from 0 to 8, where 0 points indicates a maximum dependence and 8 points indicates total independence) [20].
  - Cognitive capacity. The Pfeiffer test that evaluates the existence of cognitive deterioration (from 0 to 10 errors, where at 0–2 there is no deterioration, 3–4 mild errors, 5–7 moderate and 8–10 is important) [20].
  - Physical activity. Reduced Version in Spanish of the questionnaire of physical activity of Minnesota (VREM). It asks about the physical activity carried out over the past month and classifies individuals as low, moderate or high level, based on the quantity of METS (caloric expense) spent on a weekly basis doing physical activity [22].
- Other variables:
  - Affiliation data; inclusion date; anthropometric measurements (weight, height, body mass index); pathological history; usual pharmacological treatment; subjective assessment by the participant of the sensory organs (vision and hearing): normal, unilateral and bilateral impairment; need or no need of technical assistance for walking; history of walking rehabilitation treatment over the past year (Yes/No).

And only for the intervention group: acceptance of the console and the level of satisfaction with the exercise program carried out with the Wii, via two tests that were created for these purposes to evaluate acceptance and satisfaction in an on-going manner, from 0 to 10 points.

### Follow up

Throughout the study and using quarterly telephone calls, the falls occurring in the two groups were determined (if occurring or not and their number), as well as their consequences. The clinical histories are also reviewed by collecting information on hospital stays and urgent care visits throughout the entire study period. The date and cause of quitting the study was also included as well as the loss of follow-up information and its causes. Loss is considered to occur when the person voluntarily leaves the study, when training attendance is less than 80 %, or with the exit or impossibility of completing the follow up visits due to health reasons or other causes.

### Recruitment

The selection of the sample was carried out via telephone calls in which participants were requested to participate in the study; the participant list was obtained by filtering the population assigned to each health center by age using a computer system. Volunteers were also accepted who, based on the inclusion criteria, were interested in the study after seeing informative signs that were hung in the five primary care centers or via family members or acquaintances.

Specific agendas were created in each center to program the study visits. Potential candidates who were selected were scheduled for an initial visit in order to assess whether or not they complied with the inclusion criteria and if so, they were explained the study objectives, given an informative sheet for participants, and were asked to sign the informed consent.

**Fig. 1:**
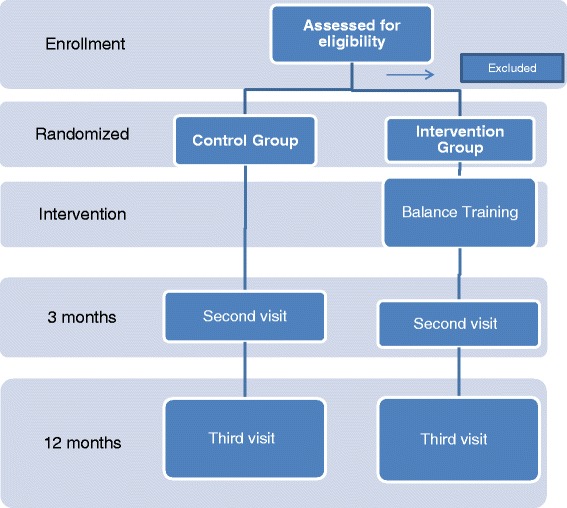
Flowchart study design

All of the participants were scheduled for 3 visits in their primary care center for the entire study period, conducted by nursing personnel who were trained to carry out the test in a homogenous manner (Fig. 1).

### Randomization

At the initial visit, having revised the inclusion/exclusion criteria and signing the informed consent, each participant was assigned an identification code. This code contained 4 digits: the first referred to their primary healthcare center and the rest were sequential numbers. Participants were randomly assigned to the control or intervention group based on whether or not their code was odd or even (evens were the control group and odds were the intervention group).

### Intervention

The control group only received the standard practice carried out in a primary care center, consisting of systematically asking the population if they exercise. For affirmative responses, the maintenance of the activity was reinforced and if the answer was negative, personalized advice was offered, depending on their age, physical condition and accompanying pathologies. In fragile elderly, they were asked about falls or their risks of suffering them.

The intervention group, in addition to receiving the same standard practice, also carried out balance training using the Nintendo™ Wii console and its balance board. Eight out of the nine balance exercises were used from the Wii Fit™ game (balance bubble, soccer heading, ski jump, table tilt, ski slalom, penguin slide, snowboard slalom, tightrope walk); the Zazen exercise was excluded as it is carried out in a seated position on the board, and since not all participants could complete this exercise due to the difficulty in sitting and standing up from the floor [23]. Participants completed 2 sessions per week for 30 min each, over a period of three months and were distributed in groups of four individuals carrying out the exercises at once. The groups were led by monitors who had received standardized training.

During the training sessions, each participant was barefoot on the balance board, carrying out the different exercises in the balance area of the Wii Fit™ game, according to the indications made on the screen. The monitor was in charge of handling the consoles, collecting data and establishing the order and time of the exercises so that in each session, all of them were carried out. The number of repetitions of each exercise varied depending on the skill of the participants but the total time dedicated to each exercise was the same for all group participants. Each group was always monitored by the same individual and therefore, each participant had a reference nurse and reference monitor throughout the study to prevent observer bias.

The intervention was considered to be completed if the participant attended at least 80 % of the sessions.

The second visit was carried out after the 3 month period (upon completing the intervention for the intervention group) and the third was carried out a year after beginning the study (9 months post-intervention), thus, the two groups participated in the study for one year. The variables collected in each visit are described in Table 1.

**Table 1:** Variables collected in each visit by groups

	Visit 1	Visit 2	Visit 3
		3 months post intervention	A year after the study
Intervention	● % balance Wii	● % balance Wii	● % balance Wii
	● Tinetti test	● Tinetti test	● Tinetti test
	● One foot Stationary	● One foot Stationary	● One foot Stationary
	● FES-I	● FES-I	● FES-I
	● Falls and consequences the year previous to the study	● Falls and consequences	● Falls and consequences
	● VREM	● Satisfaction and acceptability of the console	
	● Pfeiffer		
	● Lawton & Brodi		
Control	● % balance Wii	● % balance Wii	● % balance Wii
	● Tinetti test	● Tinetti test	● Tinetti test
	● One foot Stationary	● One foot Stationary	● One foot Stationary
	● FES-I	● FES-I	● FES-I
	● Falls and consequences the year prior to the study	● Falls and consequences	● Falls and consequences
	● VREM		
	● Pfeiffer		
	● Lawton & Brodi		

### Plan of analysis

Having refined the data, a univariate descriptive analysis was carried out to differentiate the control group from the intervention group, the frequency and percentage of the qualitative variables and the mean and standard deviation or median and interquartile range or the quantitative variables. The comparison of proportions was carried out using the Chi-square test or the Fisher exact test and the comparison of means with the Student *t* test (2 means) or an ANOVA (>2 means). When the premises for application of the indicated tests were not met, non-parametric tests were used. To evaluate the effectiveness of the intervention with the console (the primary objective) the values of the Tinetti balance test were compared for the two groups (control vs. intervention) using the Student *t* test (parametric test) if the distribution of these results was normal or with the Mann-Whitney *U* test (on-parametric test) if the distribution was not normal. This was carried out via analysis of intent to treat, a method that may be used in the analysis of all patients, regardless of whether or not they completed the study and later, the same analysis may be used on only those patients completing the study (analysis by protocol). Finally, the results of these two methods were compared. The percentage of yearly falls were compared for the two groups using the Chi square test and the mean number of falls with the Student *t* test or Mann-Whitney *U* test (first secondary objective). The same tests were used to compare the values of the FES-I test (third secondary objective). The correlation between the balance offered by the Wii console and that offered by the Tinetti tests and the one foot stationary test was measured with the Pearson correlation coefficient, since these are quantitative variables, and in a graphic manner via a scatter plot (second secondary objective). The responses of the two questionnaires evaluating the acceptability of the console and the degree of satisfaction were described using the mean and standard deviation or the median and the interquartile range, since these are two quantitative variables (fourth secondary objective). The differences between means, medians and proportions were also examined at the start of the study, after three months and one year after the intervention (pre vs. post intervention), that is, comparisons for paired data via the McNemar test, the Student *t* test for paired data or the Wilcoxon sum rank test. A multiple logistics regression model was fit with the control/intervention group as the dependent variable and with the possible predictive independent variables and those that were considered to be clinically relevant. The Stata version 11 statistics program was used to analyze the data.

### Ethics

The confidentiality and anonymity of the participant data were guaranteed both for the implementation stage as well as for any presentations or publications that may be derived from the same. An informative sheet was distributed to the participants and their informed consent was requested in writing. The research protocol has been reviewed and approved by the Committee of Ethics and Clinical Research of the Institute of Primary Care Research (IDIAP) Jordi Gol (Barcelona, Spain).

## Discussion

The project assesses the effectiveness of an intervention carried out to improve balance and subsequently prevent falls in the elderly. The greatest contribution of this intervention would be the achievement of improved balance in a relatively economic (no need for specialized healthcare personnel, avoiding displacements) and enjoyable manner, which may strengthen its sustainability in the home while at the same time, facilitating the relationship between different intergenerational family members (grandparents and grandchildren sharing the same console games). It may also be possible to carry out this program in social healthcare centers or in geriatric residences, since the only instrument necessary is the console which is currently available at an affordable cost. Healthcare savings derived from the prevention of falls in the elderly would be considerable [24, 25] and are a vital necessary for social welfare, particularly in light of the current economic situation with the resulting budgetary restrictions. Obviously, improved balance contributes to a decreased fear of falling and thereby fosters the autonomy of the individual, increasing the quality of life of the elderly in our society [26].

As for the measurement of the main results variables, some authors have suggested that the Tinetti test is not sensitive to the changes in balance suffered by patients, however, the instruments that measure time (one foot stationary test or the Timed Up & Go) seem to be more sensitive to these changes [27], therefore we decided to apply both tests to the balance assessment. On the other hand, in the majority of the literature, it was found that the console’s balance test was based on the center of gravity; in our case, we chose the console’s lame duck test since it is similar to the one foot stationary test and therefore, it would be possible to subsequently make a correlation between them.

The combination of elderly individuals and new technology may suggest difficulties when carrying out the intervention but studies that have been published on the topic [28] and the unpublished conclusions from a pilot study carried out by our research team with 20 patients from the Socio-Healthcare Interdisciplinary Functional Unit (UFISS) of Geriatrics at the Hospital Germans Trias i Pujol (Badalona, Spain) conducted to assess project viability (acceptance and possibility of implementation), suggest that this difficulty is minimal thanks to the positive characteristics of the console.

Potential selection bias that may occur when selecting volunteers based on informative campaigns was quantified by including a variable to identify how the patient was recruited (from their general care physician or nurse or other information sources) and taking this into consideration during the statistical analysis process. On the other hand, the random assignment to the study groups (intervention or control) improves the internal validity of the study as it ensures the comparability between the groups. Participation of the more active patients and volunteers in all cases may represent a problem in the external validity or representativeness of the geriatric population attended to in the healthcare centers, but we do not believe that this would invalidate the results.

The intervention characteristics prevent the application of masking techniques except in the results analysis phase where the analyst is blind to the intervention assignment.

The intervention is carried out in a group manner but the assignation is carried out on an individual basis given the differences of the populations between the primary healthcare centers which prevents them from being compared. We believe that there is no contamination since this is a very specific intervention. It was decided not to use specialize monitors (physiotherapists, physical education or health teachers) in order to highlight ease of intervention in regards to the applicability and sustainable nature in the home environment and to support the external validity of the study.

This study has been planned as a group intervention with a monitor to facilitate the completion of the same, in the least amount of time possible, but it is anticipated that it would be equivalent to carrying out these activities at home and alone. Considering the recreational aspect of the console, we anticipate that the motivation to continue improving one’s physical condition in an enjoyable manner will contribute to the sustainability and follow up of the intervention at home.

Currently, gamification [29] projects are being tested in order to obtain better results in health and physical activity maintenance; we believe that the results obtained from our study may offer knowledge in this field as well as an assessment of their applicability in the social healthcare environment.

## Abbreviations

ICASS: Institut Català d’Assistència i Serveis Socials
